# Antibiotic prescriptions for children younger than 5 years with acute upper respiratory infections in China: a retrospective nationwide claims database study

**DOI:** 10.1186/s12879-021-05997-w

**Published:** 2021-04-12

**Authors:** Fengxia Xue, Baoping Xu, Adong Shen, Kunling Shen

**Affiliations:** 1 National Clinical Research Center for Respiratory Diseases, Department of Respiratory Medicine, Beijing Children’s Hospital, Capital Medical University, National Center for Children’s Health, Beijing, 100045 China; 2 Beijing Key Laboratory of Pediatric Respiratory Infection Diseases, Key Laboratory of Major Diseases in Children, Ministry of Education, National Clinical Research Center for Respiratory Diseases, National Key Discipline of Pediatrics (Capital Medical University), Beijing Pediatric Research Institute, Beijing Children’s Hospital, Capital Medical University, National Center for Children’s Health, Beijing, 100045 China

**Keywords:** Children, Upper respiratory infections, Antibiotic prescription, China

## Abstract

**Background:**

In China, there were few studies to estimate antibiotic use for children with upper respiratory infections at the national level. The aim of this study was to describe the antibiotic prescribing practice for children aged < 5 years old with upper respiratory infections (URIs) using a nationwide claims database.

**Methods:**

This was a retrospective cross-sectional study using a sampled database from the China Health Insurance Research Association (CHIRA). Study subjects included children younger than 5 years with outpatient visits in 2015 that resulted in a diagnosis of a upper respiratory infection. We calculated the percentage of visits who received antibiotics, the proportion of injection formulations, the percentage of combined antibiotics and the proportion of each antibiotic class. The patterns of antibiotic prescription were also described by medical institution type, city level and geographical region.

**Results:**

Among the 92,821 visits, 27.1% were prescribed antibiotics, of which 27.0% received injection formulations. The rate of antibiotic prescribing varied by age group (*P* < 0.001), with the lowest (16.0%) in infants and the highest in patients at age 3 to < 4 years (29.9%) and age 4 to < 5 years (32.5%). The Midwestern region, underdeveloped cities and low-level hospitals represented relatively higher rates of prescribing antibiotics (*P* < 0.001) and higher proportions of injection dosage forms (*P* < 0.001). The most 3 common antibiotic classes prescribed of all visits with antibiotic prescriptions were the third-generation cephalosporins (34.9%), macrolides (24.3%), and the second-generation cephalosporins (23.3%).

**Conclusions:**

In mainland China, the overall rate of antibacterial prescribing and the proportion of injection formulations prescribed in children under 5 years with URIs were at a low level, but still higher in underdeveloped regions and cities. Moreover, the overuse of the second and third generation cephalosporins, macrolides, remains a serious issue. Further efforts should be focused on reducing those non-first-line antibiotic prescribing and narrowing the gaps among regions and cities.

## Background

Antibiotics are one of the most common medications prescribed for outpatients [1–3], and Upper Respiratory Infections (URIs) account for approximately 50–70% of total antibiotic prescriptions, even though most cases are of viral origin [4–7]. A prior study in US estimating potential rate of inappropriate outpatient antibiotic use had relied on estimates of bacterial prevalence and concluded that more than 50% of antibiotics for URIs were unnecessary [8]. The latest study of China reported that [9], the proportion of inappropriate antibiotic prescribing in China is higher than that in the US and UK. Fortunately, in the past 10 years, the overall decline in the use of antibiotics was found both in US, European countries, and China [10–14]. However, compared with European and American countries, the proportion of broad-spectrum antibiotics (second- and third generation cephalosporins and azithromycin, etc.) was still higher in China [15].

Over and inappropriate use of antibiotics, especially the broad-spectrum antibiotics, not only contributes to the financial burden on health services but also exposes patients to adverse events. More importantly, it is a major contributor to emerging antimicrobial resistance [4], which poses a substantial threat to global public health [16] and the UN General Assembly has recognized antimicrobial resistance as a global priority health issue. To response, China has taken many actions to curb the development of antimicrobial resistance. During the period of 2011–2014, the National Health and Family Planning Commission launched a 3-year Special Antimicrobial Stewardship Campaign nationwide. In 2016, the National Action Plan on Bacterial Resistance was formulated responding to WHO’s request for the “Global Action Plan on Antimicrobial Resistance”. Subsequently, in 2017, with the goal of strengthening the management of antimicrobial use in children, the China Children Action Plan for Rational Use Antimicrobials was released. However, the antimicrobial resistance in key pathogens remains high in China and there were increasing rates of resistance to carbapenems in clinical isolates of *Escherichia coli* and *Klebsiella pneumoniae* during 2014–2017 [17].

The surveillance of antibiotic use is necessary to formulate targeted strategies for prevention and containment of antimicrobial resistance. To date, in mainland China, there are mainly 2 nationwide network systems to monitor antibiotic use and antimicrobial resistance, such as China Antimicrobial Surveillance Network (CHINET) and China Antimicrobial Resistance Surveillance System (CARSS). However, the available national surveillance data samples are largely from adult patients, which might not truly reflect the situation in the pediatric settings. In addition, previous studies on pediatric antibiotic use for URIs were limited to selected health facilities or certain areas in China [18, 19], which might not reflect antibiotic use across the whole country. And thus, we used a nationwide claims database to investigate the antibiotic prescribing practice for pediatric patients with URIs in ambulatory settings.

## Methods

### Data source

**Fig. 1:**
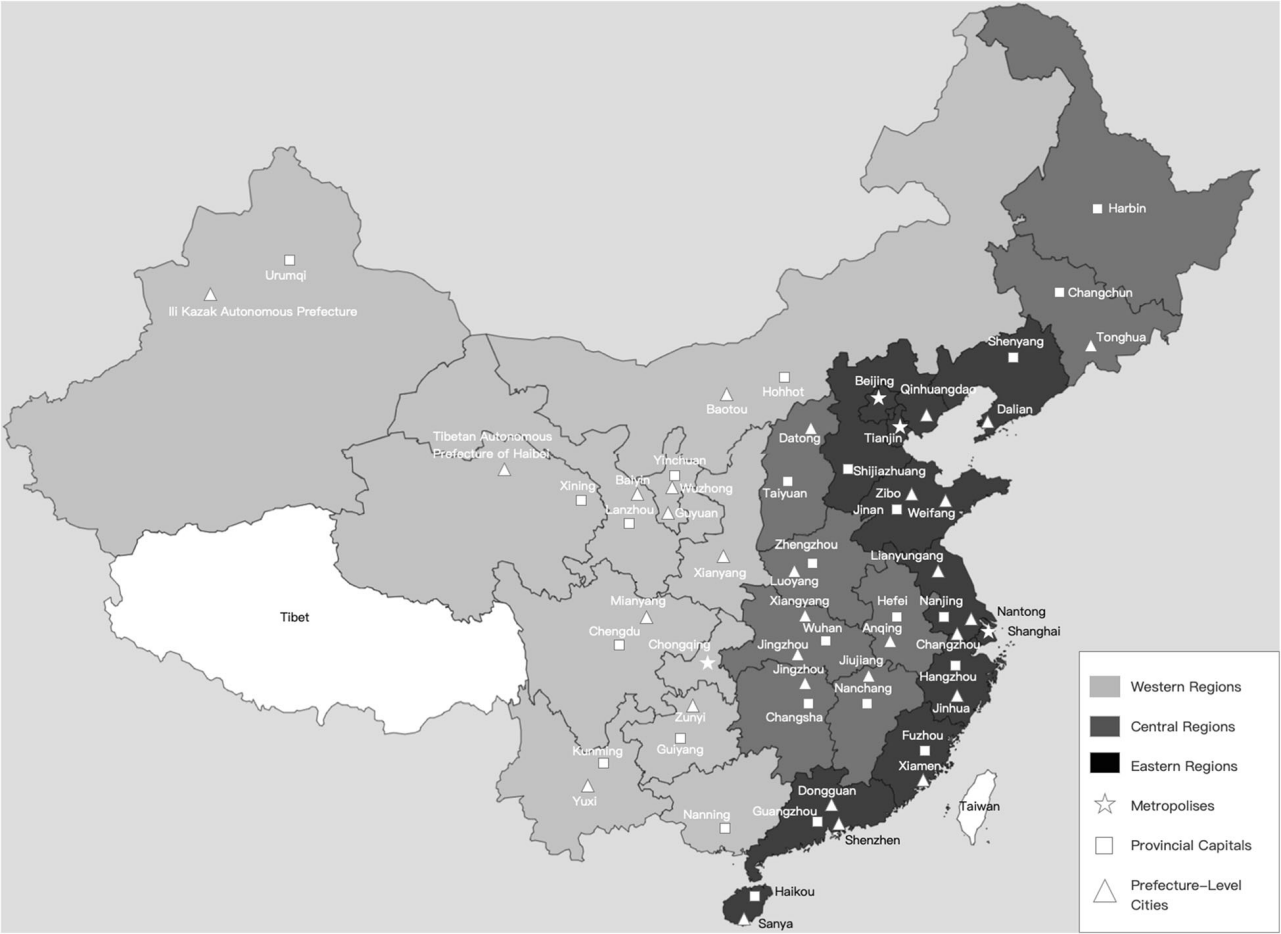
The distribution of sample cities in the CHIRA database of 2015 in mainland China

This was a retrospective, cross-sectional study using a sample from a national insurance reimbursement claims database managed by the China Health Insurance Research Association (CHIRA). The CHIRA database was established in 2007 and is managed by the Ministry of Human Resources and Social Security of the People’s Republic of China. As an administrative database, the CHIRA retrospectively and continuously collects reimbursement records of outpatient, emergency, and hospitalization of insured patients in that year since 2013. Those patients were covered by the public health insurance of China, including the Urban Employee Basic Health Insurance, the Urban Resident Basic Medical Insurance, and the New Cooperative Medical Scheme. In 2013, 95.1% of the population in mainland China covered by public medical insurance [20]. In our study, data from the CHIRA were subjected to a two-stage design. In the first stage, convenience sampling was used to include four metropolises directly under the Central Government (Beijing, Shanghai, Tianjin, and Chongqing), most provincial capital cities and other prefecture-level cities (Fig. 1) for which the Medical Insurance Bureau was able and willing to provide electronic hospital record data. In the second stage, systematic random sampling was used to extract 2% of beneficiaries.

Anonymized information on patients (age, sex), clinical diagnoses, medical institutions (type of institution, city and region located), medical procedures, prescribed drugs (generic name, prescription date, formulation, fee) were extracted. All information generated during the same visit could be linked by a unique identifier consisting of the hospital code, patient identification number and the date of visit. This study was performed in strict accordance with the human subject protection guidance of Ministry of Science and Technology of China, and was approved by the Ethics Committee of Beijing Children’s Hospital, Capital Medical University. Informed consent was not required for the use of encrypted retrospective information.

### Study design and definitions

Study subjects included children younger than 5 years with outpatient visits between 1 January and 31 December 2015 that resulted in a diagnosis of a URI. Diagnoses of URIs were identified according to the diagnosis description in the Chinese version of International Classification of Diseases, Tenth Revision (ICD-10), codes J00–J06 [21] (J00 = acute nasopharyngitis [common cold]; J01 = acute sinusitis; J02 = acute pharyngitis; J03 = acute tonsillitis; J04 = acute laryngitis and tracheitis; J05 = acute obstructive laryngitis [croup] and epiglottitis; J06 = acute upper respiratory infections of multiple and unspecified sites). Visits younger than 28 days old were excluded considering the uncertainty in management due to possible congenital factors. We also excluded visits with incomplete electronic health records.

Therapeutic drug classes were identified according to the WHO Anatomical Therapeutic Chemical (ATC) classification system (version 2020) [22]. Antibiotics were grouped into penicillins (J01CA, J01CE, J01CF, J01CG, and J01CR), first-generation cephalosporins (J01DB), second-generation cephalosporins (J01DC), third-generation cephalosporins (J01DD), fourth-generation cephalosporins (J01DE), macrolides and lincosamides (J01FA and J01FF), and others.

Demographic data including sex, age, and geographical location were collected. We divided the enrolled children into five age groups: 0 to < 1 year; 1 to < 2 years; 2 to < 3 years; 3 to < 4 years; and 4 to < 5 years. Based on the geographical location, medical institutions were separated into the eastern, central, and western regions [23]. Cities were classified from level 1 to level 4 according to the City Socioeconomic Survey conducted by the National Bureau of Statistics of China (version 2016) [24] (level 1 = metropolises; level 2 = economically developed provincial capitals; level 3 = some economically underdeveloped provincial capitals and developed prefecture-level cities; level 4 = some economically underdeveloped prefecture-level cities). Based on certification levels, medical institutions were divided into tertiary hospitals, secondary hospitals, and primary care facilities [25].

### Statistical analyses

In this study, the antibiotic prescriptions were analyzed using every outpatient visit as the unit. The multiple prescriptions and diagnoses for the same patient at the same time in the same hospital were considered as one visit. Evaluation indicators were as follows: (1) the proportion of visits with an antibiotic prescribed; (2) the proportion of injectable antibiotics prescribed; (3) the proportion of combined antibiotics prescribed;(4) the proportion of antibiotics prescribed in each ATC category. To investigate the antibiotic use in detail, we performed a comprehensive analysis according to the geographic regions, the level of the cities and the level of medical care institutions.

Prescription rates, the proportions of injectable antibiotics and combined antibiotics were compared across subgroups using the Chi-Square test. *P*-values less than 0.05 were considered statistically significant. All data were exported into an Excel spreadsheet (Microsoft). Descriptive analyses were conducted using Excel (Microsoft). SPSS25.0 and GraphPad Prism 8 were used for statistical analysis and plotting, respectively.

## Results

Table 1 showed the demographics of outpatients younger than 5 years with URIs. In total, there were 92,821 sampled ambulatory care visits for URIs from 59 cities included in the analysis. 73,638 outpatient visits were from the east region of China, which accounted for 79.3% of all visits. There were 41,094 (44.3% of all visits) visits presented in metropolises, and 38,275 (41.2% of all visits) visits presented in provincial capitals.

**Table 1:** Demographics of outpatient visits younger than 5 years with URIs

	**Total**	**Age (years)**					**Region**		
		<1	1 to<2	2 to<3	3 to<4	4 to<5	East	Central	West
**Visits (%)**	92821 (100)	4798 (5.2)	20625 (22.2)	15983 (17.2)	26575 (28.6)	24840 (26.8)	73638 (79.3)	1229 (1.3)	17954 (19.3)
	**City level**				**Medical institution type**				
	Level 1	Level 2	Level 3	Level 4	Tertiary	Secondary	Primary	Uncertain	
**Visits (%)**	41094 (44.3)	38275 (41.2)	6699 (7.2)	6753 (7.3)	35717 (38.5)	18622 (20.1)	36776 (39.6)	1706 (1.8)	

Table 2 showed the frequency and proportion of antibiotic prescriptions in visits. Overall, antibiotics were prescribed in 27.1% of the outpatient visits for URIs (25,193 of 92,821 visits), of which 27.0% (6814 of 25,193 visits with antibiotic prescriptions) were injection formulations. Of the 25,193 visits receiving antibiotics, 5.7% (1430) received 2 or more antibiotic agents at the same visit. There was a trend towards increasing prescription rate of antibiotics with increasing age groups [(16.0% (767 of 4798 visits)] for children in the 0- to < 1-year-old age group, 20.4% (4205 of 20,625 visits) for the 1 to < 2-year-old group, 26.3% (4201 of 15,983 visits) for the 2 to < 3-year-old group, 29.9% (7951 of 26,575 visits) for the 3 to < 4-year-old group and 32.5% (8071 of 24,840 visits) for the 4 to < 5-year-old group; data were not shown]. The antibiotic prescribing practices varied by regions (*p* < 0.001), and the rate of prescribing antibiotics and the proportion of injection formulations were highest in central region, at 36.9 and 64.5%, respectively, followed by the west (32.8, 36.4%) and east (25.6, 23.2%) of China. Compared with other cities, visits from the fourth-tier cities received more antibiotics and injections (39.5 and 56.9%, respectively, *p* < 0.001). Among medical institutions, healthcare providers in primary care facilities tended to prescribe more antibiotics and injection dosage for URIs than those in tertiary hospitals (*P* < 0.001).

**Table 2:** Frequency and proportion of antibiotic prescriptions in visits for various subgroups

	Visits with antibiotics prescribed, n(%)*^1^	***P*** value	Visits with injection form prescribed, n(%)*^2^	*P* value	Visits with combined antibiotics prescribed, n(%)*^3^	*P* value
**Total**	25,193 (27.1)		6814 (27.0)		1430 (5.7)	
**Region**		*P* < 0.001		*P* < 0.001		*P* < 0.001
East	18,847 (25.6)		4374 (23.2)		861 (4.6)	
Central	454 (36.9)		293 (64.5)		45 (9.9)	
West	5892 (32.8)		2147 (36.4)		524 (8.9)	
**City level**		*P* < 0.001		*P* < 0.001		*P* = 0.008
Level 1	10,617 (25.8)		2246 (21.2)		598 (5.6)	
Level 2	9932 (25.9)		2479 (25.0)		592 (6.0)	
Level 3	1976 (29.5)		571 (28.9)		80 (4.0)	
Level 4	2668 (39.5)		1518 (56.9)		160 (6.0)	
**Medical institution**		*P* < 0.001		*P* < 0.001		*P* < 0.001
Tertiary hospitals	9104 (25.5)		2242 (24.6)		300 (3.3)	
Secondary hospitals	4921 (26.4)		1569 (31.9)		227 (4.6)	
Primary care facilities	10,731 (29.2)		2965 (27.6)		894 (8.3)	

**Fig. 2:**
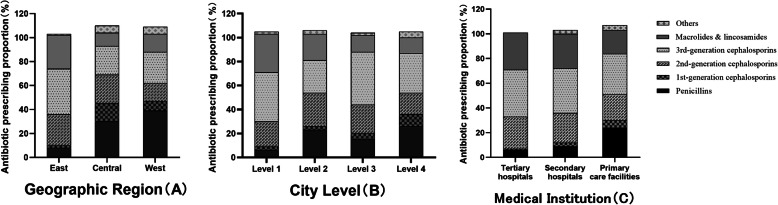
Types of antibiotics prescribed for various subgroups. (**a** Geographic Region; **b** City Level; **c** Medical Institution). The reason why the column is higher than 100% is that the child may have been prescribed 2 or more antibiotic agents in the same visit

*^1^**% for visits with antibiotics prescribed** was calculated by dividing the number of visits with antibiotics prescribed by the total number of visits and multiplying by 100; *^2^**% for Visits with injection dosage form prescribed** was calculated by dividing the number of visits with injection dosage form prescribed by the total number of visits in which antibiotics were prescribed and multiplying by 100; *^3^**% for Visits with combination antibiotics prescribed** was calculated by dividing the number of visits with combination antibiotics prescribed by the total number of visits in which antibiotics were prescribed and multiplying by 100;

Figure 2 showed the types antibiotic prescriptions based on ATC classification. Third-generation cephalosporins (J01DD) were the most common group of antibiotics prescribed (34.9% of all visits with antibiotics prescriptions, 8790/25193), followed by macrolides and lincosamides (J01FA and J01FF; 24.3%, 6133/25193), second generation cephalosporins (J01DC; 23.3%, 5876/25193). Such a prescribing pattern was more obvious in the eastern region, economically developed cities, tertiary and secondary hospitals. Of 25,193 visits with antibiotic prescriptions, only 3937 (15.6%) were prescribed penicillins (J01CA, J01CE, J01CF, J01CG and J01CR). In Central and Western China, penicillins (J01CA, J01CE, J01CF, J01CG and J01CR) were most used, accounting for 29.5% (134/454) and 38.7% (2279/5892), respectively. The top 10 antibiotic agents appeared in Table 3. Cefixime (20.4%, 5146/25193), azithromycin (15.4%, 3881/25193), and cefaclor (12.7%, 3186/25193) were the 3 most frequently dispensed, accounting for half of all visits with antibiotic prescriptions cumulatively.

**Table 3:** The top 10 antibiotic agents prescribed

Agents	Antibiotic use, n (%*)	Cumulative antibiotic use, n(%)
**Cefixime**	5146 (20.4)	5146 (20.4)
**Azithromycin**	3881 (15.4)	9027 (35.8)
**Cefaclor**	3186 (12.7)	12,213 (48.5)
**Amoxicillin Clavulanate Potassium**	2017 (8.0)	14,230 (56.5)
**Amoxicillin**	1361 (5.4)	15,591 (61.9)
**Cefdinir**	1326 (5.3)	16,917 (67.1)
**Cefuroxime**	1215 (4.8)	18,132 (72.0)
**Clarithromycin**	1139 (4.5)	19,271 (76.5)
**Cefprozil**	824 (3.3)	20,095 (79.8)
**Ergomycin**	801 (3.2)	20,896 (82.9)

* % was calculated by dividing the number of visits with specific antibiotic agent by the total number of visits in which antibiotics were prescribed and multiplying by 100

## Discussion

As early as 2000, the Respiratory Group of Pediatrics Society, Chinese Medical Association have published the guideline for rational use of antibiotics for acute respiratory infections [26]. Furthermore, in 2018, the Respiratory Society of Chinese Medical Association released general guidance for URIs and revised in 2020 [27], indicating that the management of the URIs should focus on relieving symptoms rather than prescribing antibiotics, unless suspected of having bacterial infection. Antibiotics recommended for treating URIs were penicillins, first-generation cephalosporins, macrolides and quinolones. Penicillins were recommended as the first-line therapy for children. At present, quinolones are not recommended as first-line therapy for children in China. In this analysis of a large, nationally representative database of outpatient prescriptions, we found that the 3 most prescribed antibacterial agents were cefixime, azithromycin, and cefaclor, which was not in accordance with the guideline recommendations in China.

Currently, the percentage of visits with an antibiotic prescribed is a main indicator for assessing and supervising antibiotic use [28]. In 2011, the European Surveillance of Antimicrobial Consumption project (ESAC) [29] recommends that the percentage of antibiotics prescribed for outpatients > 1 year with acute URIs and acute tonsillitis should be limited to 0–20%. During the 2011–2014, the nationwide special rectification campaign of China [30] stipulating that the prescription rate for outpatients in general hospitals and pediatric hospitals should not exceed 20 and 25%, respectively. In our study, we estimated that 27.1% of pediatric outpatients under 5 years with URIs received antibiotic prescriptions, which was slightly higher than the rate recommended by the ESAC [29] and the Ministry of Health of the P.R. China [30] but lower than the previous studies of China. For example, Yuan. et al. [19] reported that 90.45% pediatric outpatient visits for suppurative tonsillitis resulted in antibiotic prescriptions, 78.77% for pharyngitis, and 52.25% for other URIs in 2010–2011. In a 2017 meta-analysis in China [31], 89.2% of children received antibiotics for acute URIs in the past 16 years. The reduction in antibiotic prescription rate was largely attributed to the antibiotic stewardship campaigns of China and some evidence have confirmed its positive impact [12, 32]. Since we only had collected the data on 2015, the trend in antibiotic use and the effects of the antimicrobial stewardship program on antibiotic prescribing could not be evaluated directly in our study.

In other countries, there also showed high antibiotic prescription rates for URIs. In the United States, Fleming-Dutra. et al. [4] reported that of all the sampled ambulatory care visits for the population younger than 19 years, 84.7% were associated with antibiotic prescriptions for sinusitis, 56.2% for pharyngitis, and 21.2% for viral URIs, which was similar with the findings of Kronman. et al. in 2014 [8]. The differences between these studies in US and ours are probably resulted by the fact that we did not separate pharyngitis and sinusitis from URIs. Also, the discrepancy of results could be explained by the different regions and populations. And, Zhao. et al. [33] had reported that children aged 6–17 years had the higher antibiotic prescription rate for URIs than preschoolers. Compare with other foreign studies, 27.1% of visits resulted in antibiotic prescriptions, considerably lower than reported in the South Korea (58.7%) [34] but higher than reported in the Spain (< 20%) [35].

In our study, approximately one-third of the prescribed antibiotics were in injection formulations. According the Guiding Principles of China [36], most patients who require antimicrobial drugs for mild-to-moderate infections should be treated orally instead of intravenously or intramuscularly. Moreover, injections expose patients to unnecessary drug-related side effects, substantial financial burden, and medical risks, such as bloodborne diseases. It is gratifying that, compared with previous studies in China, our result is substantially lower than the percentages of injections reported in Yuan’s (78.22% for suppurative tonsillitis, 60.58% for pharyngitis, and 16.47% for other URIs) and Zhang’s (41%) studies [18, 19]. Furthermore, the percentage of injections used maybe overestimated in our study, because the fact that there are more visits related to this administration form, while for oral antibiotic use there is only one or maximum plus one follow-up visit.

In our study, the most common antibiotics prescribed (79%) were third generation cephalosporins, macrolides and second generation cephalosporins, which is consistent with prior studies in China [15, 19, 37] and Japan [38, 39]. In fact, penicillins were the first choice recommended for URIs by the guidelines of China [26, 27]. Recommendations from ESAC indicated that beta-lactamase-sensitive penicillins (J01CE) should account for 80–100% of antibiotics used for URIs [29]. However, this proportion for all penicillins (J01CA, J01CE, J01CF, J01CG and J01CR) was only 15.6% in China. On the contrary, a cross-national analysis on antibiotic use in children of 6 countries found that [40], in the United States, South Korea, Italy, Spain, and Norway, penicillins were the most common prescribed antibiotics. In US and Norway [40, 41], if a penicillin was prescribed for pediatrics, the narrow-spectrum penicillins were commonly used. According to the Chinese Pharmacopeia Guidelines for Clinical Drug Use, penicillins require skin tests before use in China, which largely limits the use of penicillins in our country.

Compared with the top 10 antibacterial agents in pediatrics worldwide [42], the proportion of broad-spectrum antibacterial used was significantly higher in our study, especially in the eastern region, economically developed cities, tertiary and secondary hospitals. However, compared with narrow-spectrum antibiotics, broad-spectrum antibiotics were unable to provide better clinical outcomes and were associated with higher rates of adverse events for children with acute respiratory tract infections [43]. More broad-spectrum antibiotics prescribed may be due to physicians’ uncertainty about etiology, insufficient knowledge of bacterial resistance, driven by economic interests and urging of parents [44]. Therefore, to restrict the use of broad-spectrum antibiotics, it is necessary to strengthen continuous medical education on the management of childhood common infections and supervise the compliance of antimicrobial stewardship recommendations.

Our analysis also showed that pediatric visits in midwestern China and underdeveloped cities received more antibiotics and injection dosage forms. To some extent, such differences were related to the imbalanced economic development and discrepancies in healthcare education resources. Also, studies in Japan [39] and Korea [34] suggested that health facility level are associated with antibiotic prescriptions in agreement with our results. To narrow the gaps among regions and cities and promote the rational use of antibacterial drugs, efforts are urgently needed to strengthen the supervision and intervention of antimicrobial prescribing. Establishing a nationwide pediatric surveillance network is the key to monitoring antibiotic use in children. Other studies have shown that the characteristics of physicians, such as specialty, education level and seniority, have a significant influence on the prescribing of antibiotics [45–48], but relevant evidence is limited in China. Thus, further study should focus on the effect of provider characteristics on antibiotic prescribing.

To our knowledge, in mainland China, this study is the first to describe antibiotic prescriptions for children with URIs using a claim database covering almost all provinces of China. However, there were also some limitations. First, data from rural areas were not available in the 2015 CHIRA database, therefore, it was not representative of rural China. Second, in this study, each visit for an infection was the unit of analysis and not each infection episode. Thus, antibiotic prescriptions within the same infection episode were captured multiple times. Third, the diagnosis of URIs identified was failed to eliminate other co-infections. Therefore, we could not verify that all antibiotic prescriptions in the dataset were for URIs. Fourth, it is not possible to determine how many patients were bacterial infected in our study because testing for respiratory pathogens is not routinely performed in outpatient settings. Therefore, it is urgent to promote the development of rapid pathogen tests to guide the rational use of antibiotics. Another limitation is that the CHIRA database does not collect information on vital signs, physical exam findings, drug allergy and previous medication failure of patients, which could affect physician’s treatment decisions on antibiotic selection.

## Conclusion

In mainland China, the overall rate of antimicrobial prescribing and the proportion of injection formulations prescribed in children under 5 years with upper respiratory infections were at a low level, but still higher in underdeveloped regions and cities. Moreover, the overuse of the third generation cephalosporins, macrolides, and the second generation cephalosporins remains a serious issue. Further efforts should be focused on reducing those non-first-line antibiotic prescribing and narrowing the gaps among regions and cities.

## Data Availability

The datasets generated and/or analyzed during the current study are available from the corresponding author on reasonable request.

## Abbreviations

URIs: upper respiratory infections
WHO: World Health Organization
CHIRA: The China Medical Insurance Research Association
ICD-10: International Classification of Diseases, Tenth Revision
ATC: Anatomical Therapeutic Chemical
ESAC: the European Surveillance of Antimicrobial Consumption project.
